# Ferroptosis and mitochondrial ROS are central to SARS-CoV-2-induced hepatocyte death

**DOI:** 10.3389/fcimb.2025.1625928

**Published:** 2025-08-11

**Authors:** Cintia Cevallos, Patricio Jarmoluk, Franco Sviercz, Rosa Nicole Freiberger, Cynthia Alicia Marcela López, M. Victoria Delpino, Jorge Quarleri

**Affiliations:** Universidad de Buenos Aires (UBA), Consejo de Investigaciones Científicas y Técnicas (CONICET), Instituto de Investigaciones Biomédicas en Retrovirus y Sida (INBIRS), Laboratorio de Inmunopatogénesis Viral, Buenos Aires, Argentina

**Keywords:** SARS-CoV-2, hepatocytes, mitochondrial ROS, ferroptosis, lipid metabolism, regulated cell death, transferrin receptor, COVID-19

## Abstract

**Background:**

Although COVID-19 primarily affects the respiratory tract, liver injury has been increasingly reported in infected individuals. The mechanisms by which SARS-CoV-2 induces hepatocyte damage remain poorly understood. Given the role of mitochondrial dysfunction, oxidative stress, and regulated cell death in COVID-19 pathogenesis, we investigated the impact of SARS-CoV-2 infection on hepatocytes using the Huh7.5 cell model.

**Methods:**

Huh7.5 hepatocytes were infected with either the ancestral Wuhan (Wh) or Omicron (BA.5) variant of SARS-CoV-2. Viral replication was quantified via RT-qPCR, nucleocapsid protein detection, and infectious particle titration. Mitochondrial function was assessed through mitochondrial membrane potential (ΔΨm), mROS production, and mitophagy analysis. Lipid metabolism and regulated cell death (apoptosis, pyroptosis, ferroptosis) were evaluated by confocal microscopy and flow cytometry. The role of specific cell death pathways was probed using chemical inhibitors.

**Results:**

Both SARS-CoV-2 variants efficiently infected Huh7.5 cells, with distinct replication kinetics. Infection induced mitochondrial fragmentation, elevated mROS levels, and lipid droplet accumulation. Ferroptosis was identified as a predominant mode of cell death, as evidenced by increased lipid peroxidation and the protective effect of ferrostatin-1. Expression of angiotensin-converting enzyme 2 (ACE2) and transferrin receptor 1 (TfR1), a ferroptosis marker and alternative viral entry receptor, was significantly upregulated post-infection in a variant-dependent manner. Additionally, mROS scavenging with MitoTEMPO impaired viral replication, underscoring the role of oxidative stress in the SARS-CoV-2 life cycle.

**Conclusions:**

SARS-CoV-2 disrupts mitochondrial homeostasis and lipid metabolism in hepatocytes, promoting ferroptosis as a major contributor to virus-induced cytopathology. These findings suggest that ferroptosis may play a central role in COVID-19-related liver injury and identify mitochondrial ROS and iron metabolism as potential therapeutic targets.

## 1 Introduction

COVID-19 is primarily characterized by respiratory tract infection and subsequent pulmonary involvement (Proal et al., 2023). However, extrapulmonary manifestations have also been reported, as SARS-CoV-2 exhibits tropism for various tissues (Wissler Gerdes et al., 2022). Among these, liver damage of varying severity has been observed in patients with SARS-CoV-2 infection, and clinical studies have documented the development of acute liver injury (ALI) (Dufour et al., 2022). However, the mechanisms underlying liver injury during SARS-CoV-2 infection remain unclear. Potential contributors include the virus’s direct cytopathic effects, immune response activation, and drug-induced hepatotoxicity (Fratta Pasini et al., 2021; Dufour et al., 2022).

Redox homeostasis is critical for cellular function, and viruses, including SARS-CoV-2, can disrupt mitochondrial dynamics to promote their replication. Specifically, increased mitochondrial reactive oxygen species (mROS) levels can enhance viral replication and regulate cell death pathways (Foo et al., 2022). Additionally, viral infections often interfere with lipid metabolism, and lipid droplet (LD) biogenesis has been implicated in SARS-CoV-2 replication and pathogenesis (Dias et al., 2020; D'Avila et al., 2024).

SARS-CoV-2 modulates cell death pathways, contributing to COVID-19 pathogenesis and progression (Yuan et al., 2023). Iron metabolism is disrupted in SARS-CoV-2 infection (Muhoberac, 2020), and its dysregulation can trigger ferroptosis—a form of programmed cell death driven by iron-dependent lipid peroxidation. The human transferrin receptor (TfR1, CD71) mediates Fe³^+^ uptake into cells, is upregulated during ferroptosis, and serves as a ferroptosis-specific marker. Interestingly, studies in lung cells have shown that TfR1 can mediate SARS-CoV-2 infection in an angiotensin-converting enzyme 2 (ACE2)-independent manner, allowing viral entry into tissues with low or negligible ACE2 expression (Liao et al., 2024). Ferroptosis plays a key role in COVID-19-related lung disease, and increased ROS levels may promote lipid peroxidation and ferroptosis during SARS-CoV-2 infection (Fratta Pasini et al., 2021; Qiu et al., 2024).

This study aims to elucidate the direct effects of SARS-CoV-2 infection on hepatocytes, using the Huh7.5 human hepatocyte cell line as a model. We evaluated two SARS-CoV-2 strains and found that the virus efficiently infects Huh7.5 cells, disrupts mitochondrial homeostasis and lipid metabolism to facilitate replication, and induces hepatocyte death predominantly via the ferroptosis pathway.

## 2 Materials and methods

### 2.1 Cell culture

The human Huh7.5 hepatocellular carcinoma-derived cell line was obtained from ATCC. Cells were cultured in Dulbecco’s Modified Eagle Medium (DMEM) supplemented with 10% fetal bovine serum (FBS), 2 mM L-glutamine, 100 U/mL penicillin, and 100 µg/mL streptomycin at 37°C in a 5% CO2 atmosphere. All experiments were conducted under biosafety level 3 (BSL-3) conditions at INBIRS. Biological materials were autoclaved and incinerated following institutional guidelines.

### 2.2 SARS-CoV-2 stocks and viral titration

Viral stocks were obtained, titrated, and maintained as previously described (Sviercz et al., 2024). Briefly, the ancestral Wuhan (Wh) strain was provided by Dr. Sandra Gallego (Universidad Nacional de Córdoba, Argentina). In contrast, the Omicron (BA.5) strain was isolated from a nasopharyngeal swab, characterized, propagated, and titrated in Vero cells, yielding a titer of 2.0×10^6^ plaque-forming units (PFU)/mL. Vero E6 cells (ATCC, Rockville, MD) were maintained as monolayers at 37°C in a 5% CO2 atmosphere in DMEM supplemented with 2 mM L-glutamine, 10% FBS, 100 U/mL penicillin, and 100 µg/mL streptomycin.

### 2.3 Determination of ACE2 and TfR expression in Huh7.5 cells

The cell surface expression of ACE2 and TfR (CD71) was quantified 72 hours post-infection (hpi) using flow cytometry. Cells were incubated with a rabbit polyclonal anti-ACE2 antibody (#ab272690, Abcam, UK) or a rabbit polyclonal anti-TfR/CD71 antibody (#ab84036, Abcam, UK), followed by a PE-conjugated secondary anti-rabbit IgG antibody (cat#31210, Invitrogen, US). Data were acquired using Full Spectrum Flow Cytometry Cytek^®^ Northern Lights 3000™ (Cytek Biosciences Inc.) and analyzed with FlowJo.v10.6.2 (Ashland).

### 2.4 Infection of Huh7.5 cells with SARS-CoV-2

Huh7.5 cells were seeded at 5×10^4^ cells/well in 24-well plates and infected with SARS-CoV-2 Wh or BA.5 at a multiplicity of infection (MOI) of 0.05 for 1 hour at 37°C in a 5% CO2 atmosphere. After infection, monolayers were washed three times with phosphate buffered saline (PBS) and maintained in DMEM supplemented with 2 mM L-glutamine, 10% heat-inactivated FBS, 100 U/mL penicillin, and 100 µg/mL streptomycin. Huh7.5 cells were left untreated for each condition as a negative control (mock).

### 2.5 Assessment of infection and viral replication

Viral infection and replication efficiency were assessed at 24-, 48-, and 72-hpi using the following approaches:

#### 2.5.1 Intracellular detection of SARS-CoV-2 nucleocapsid protein by flow cytometry

Cells were fixed, permeabilized, and stained with a rabbit anti-nucleocapsid antibody (clone EPR24334-118, #ab271180, Abcam), followed by a PE-conjugated secondary antibody. Fluorescence was analyzed by flow cytometry (Cytek Biosciences Inc.) to quantify infected cells with FlowJo.v10.6.2 (Ashland).

#### 2.5.2 Confocal microscopy

SARS-CoV-2-infected Huh7.5 cells at 72 hpi were fixed, permeabilized, and stained to visualize viral nucleocapsid protein for the qualitative visualization of the SARS-CoV-2 nucleocapsid protein in infected Huh7.5 cells. Images were acquired using confocal microscopy.

#### 2.5.3 Quantification of SARS-CoV-2 genomic RNA in culture supernatants

Viral RNA was extracted using the Chemagic™ Viral DNA/RNA Kit Special H96 on the automated Chemagic™ 360 instrument (PerkinElmer, Germany). RNA concentration was assessed using a NanoDrop™ spectrophotometer (Thermo Scientific™) for quality control. SARS-CoV-2 RNA was quantified by RT-qPCR using the DisCoVery SARS-CoV-2 RT-PCR Detection Kit Rox, targeting the ORF1ab and N genes. Viral load was determined by interpolating Ct values with a standard curve generated from quantified SARS-CoV-2 positive RNA controls (GISAID EPI_ISL_420600). The assay’s limit of detection (LOD) was 100 SARS-CoV-2 genomic RNA copies/mL.

#### 2.5.4 Titration of infectious viral particles

Supernatants from infected Huh7.5 cells were used to infect Vero E6 cells. After one hour of adsorption, the inoculum was replaced with DMEM containing 2% FBS. Plates were incubated for 72 hours, after which visible plaques were counted. Infectious viral titers were calculated based on plaque formation and expressed as plaque-forming units (PFU)/mL, using the formula:


PFU/mL = Number of plaques/(dilution factor × volume of inoculum).

### 2.6 Detection and scavenging of mitochondrial reactive oxygen species

mROS levels were quantified by flow cytometry (Cytek Biosciences Inc.) following staining with 5 µM MitoSOX™ Red (Thermo Fisher Scientific) for 15 minutes at 37°C in PBS. Rotenone (10 µM) was used as a positive control for mROS induction. To assess mROS scavenging, cells were pretreated with 50 µM MitoTEMPO (#SML0737, Sigma-Aldrich) for 24 hours before infection. MitoSOX™ fluorescence intensity (excitation: 510 nm, emission: 580 nm) was measured at 24-, 48-, and 72-hpi. Analysis was done with FlowJo.v10.6.2 (Ashland).

### 2.7 Measurement of mitochondrial mass and membrane potential

The mitochondrial membrane potential was assessed as previously described (Ojeda et al., 2018). At 24, 48, and 72 hpi, cells were incubated with 100 nM MitoTracker™ Deep Red (MTDR, Thermo Fisher Scientific) for 15 minutes at 37°C in appropriate culture conditions. After incubation, fluorescence intensity was analyzed by flow cytometry. A decrease in MTDR fluorescence intensity indicated mitochondrial depolarization.

Mitochondrial mass and membrane potential (ΔΨm) in Huh7.5 cells were assessed using MitoTracker™ Green FM (MTG; Thermo Fisher Scientific) and MitoTracker™ Deep Red FM (MTDR; Thermo Fisher Scientific), respectively. Briefly, cells were incubated with 100 nM MTG and 100 nM MTDR in pre-warmed complete medium at 37°C for 30 minutes, protected from light. After staining, cells were washed twice with PBS, resuspended in fresh medium, and immediately analyzed by flow cytometry (Cytek Biosciences Inc.) and FlowJo.v10.6.2 (Ashland). MTG fluorescence was used to evaluate total mitochondrial content (independent of membrane potential), while MTDR fluorescence reflected mitochondrial membrane potential.

### 2.8 Colocalization of mitochondria and lysosomes

Huh7.5 cells were infected with SARS-CoV-2 Wh or BA.5, and mitochondria-lysosome colocalization was evaluated at 24-, 48-, and 72-hpi. Mock-infected cells served as controls. Mitochondria were stained with MitoTracker™ Red CMXRos, lysosomes with LysoTracker™ Green, and nuclei with DAPI (Invitrogen). Mdivi-1 (mitophagy inhibitor) was added to the medium at a final concentration of 50 ​μmol/L for 2 ​h before infection. Cells were fixed with 4% paraformaldehyde and mounted in PBS-glycerol (9:1 v/v) with an antifade reagent for confocal microscopy analysis (Zeiss LSM 800, 63× objective). Ten microscopic fields per well were analyzed in triplicate. To quantify the degree of colocalization, we calculated the Manders’ correlation coefficient from the corresponding confocal images using FIJI software (ImageJ, National Institutes of Health, Bethesda, MD, USA) (Manders et al., 1993).

### 2.9 Lipid droplets staining

Lipid content was evaluated using Bodipy™ 493/503 staining at 24-, 48-, and 72-hpi, in the presence or absence of 10 µM Ferrostatin-1 (#SML0583-5MG, Sigma-Aldrich). Mock-infected cells, treated or untreated with Ferrostatin-1, served as controls. Cells were fixed with 4% paraformaldehyde, permeabilized with 0.3% Triton X-100, stained with Bodipy 493/503, and analyzed by confocal microscopy (Zeiss LSM 800, 63× objective). Lipid accumulation was quantified based on fluorescence intensity, analyzing ten microscopic fields per well from three wells per experimental condition.

### 2.10 Correlation between viral replication and mROS production

To assess the correlation between viral replication and mROS levels, Huh7.5 cells were pretreated with 50 µM MitoTEMPO for 24 hours, infected with SARS-CoV-2 variants, and analyzed at 24-, 48-, and 72-hpi. mROS production was quantified by flow cytometry (MitoSOX™ staining), and viral replication was assessed by RT-qPCR quantifying SARS-CoV-2 RNA in supernatants. Untreated and mock-infected controls were included for comparison.

### 2.11 Measurement of regulated cell death (apoptosis, pyroptosis, ferroptosis)

Regulated cell death (RCD) was quantified by flow cytometry using alophycocyanin (APC)-conjugated Annexin V and 7-AAD (BD Biosciences). Staurosporine (1 µM) was used as a positive control for apoptosis. Caspase-dependent and independent pathways were assessed using specific inhibitors, including the caspase-3 inhibitor Z-DEVD-FMK for apoptosis, the caspase-1 inhibitor Z-YVAD-FMK for pyroptosis, and Ferrostatin-1 (10 µM) for ferroptosis. Caspase activity was measured using the Vybrant™ FAM Caspase-3/-7 Assay Kit and the Caspase-1 Staining Kit, following the manufacturer’s protocols. RCD was analyzed at 24, 48, and 72 hpi using Full Spectrum Flow Cytometry Cytek^®^ Northern Lights 3000™ (Cytek Biosciences Inc.) and analyzed with FlowJo.v10.6.2 (Ashland).

### 2.12 Cytokine measurement

IL-1β levels were assessed using anti-IL-1β-fluorescein isothiocyanate (FITC) (JK1B-1, BioLegend, US) by flow cytometry. In parallel, IL-1β concentrations in cell culture supernatants were quantified using a commercially available human IL-1β ELISA kit (R&D Systems), following the manufacturer’s instructions. Absorbance was measured at 450 nm using a microplate reader, and cytokine concentrations were interpolated from a standard curve generated with known IL-1β concentrations.

### 2.13 Statistical analysis

Statistical analysis was performed wherever applicable. Statistical analysis was performed with one-way ANOVA. Multiple comparisons between all pairs of groups were made using Tukey’s test, and those between two groups were made using the Mann-Whitney U test. Graphical and statistical analyses were performed with GraphPad Prism 8.0 software. Each experiment was performed in triplicate (technical replicates) with different culture preparations on 2–4 independent occasions (biological replicates). Data were represented as mean ± SD measured in triplicate from three individual experiments. A p<0.05 is represented as *, p<0.01 as **, p<0.001 as ***, and p<0.0001 as ****. A statistically significant difference between groups was accepted at a minimum level of p<0.05.

## 3 Results

### 3.1 Huh7.5 cells are permissive to SARS-CoV-2 infection but exhibit variant-dependent viral kinetics

Liver damage severity may vary depending on the SARS-CoV-2 variant involved in the infection, potentially due to differences in replication kinetics and infectivity (Bartolomeo et al., 2022; Zhang et al., 2022). To assess this, viral replication and infection efficiency were evaluated at three post-infection time points -24, 48, and 72 hpi- by quantifying intracellular SARS-CoV-2 nucleocapsid protein (N) expression via flow cytometry and measuring viral load in Huh7.5 cell supernatants using RT-qPCR.

**Figure 1:**
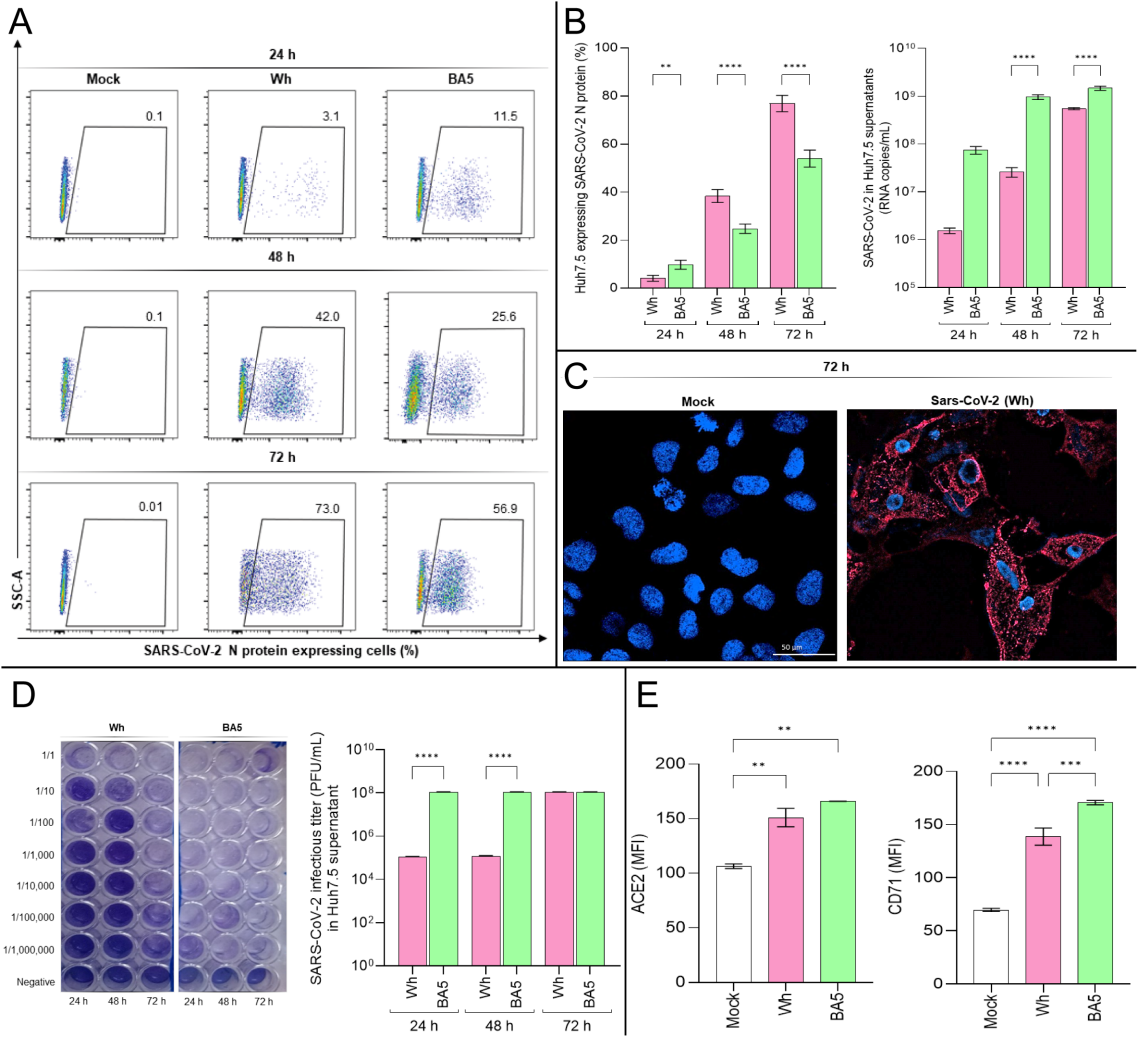
SARS-CoV-2 infection dynamics and receptor expression in Huh7.5 cells. **(A, B)** Flow cytometry analysis of intracellular SARS-CoV-2 nucleocapsid (N) protein expression in Huh7.5 cells infected with the ancestral Wuhan (Wh) or Omicron BA.5 variants at 24-, 48-, and 72-hours post-infection (hpi). **(C)** Representative confocal images of N protein expression (red) in infected cells at 72 hpi. Nuclei were stained with DAPI (blue). **(D)** Viral titers in culture supernatants determined by plaque-forming units (PFU/mL) using Vero E6 cells. **(E)** Surface expression of ACE2 and transferrin receptor 1 (TfR1) was assessed by flow cytometry at 72 hpi. Data represent mean ± SD of three independent experiments. Statistical significance was determined by one-way ANOVA with Tukey’s multiple comparisons test. **p<0.01, ***p<0.001, ****p<0.0001.

As shown in **Figures 1A, B**, N protein expression levels increased over time for both variants, but significant differences were observed between the ancestral strain (Wh) and the omicron BA.5 variant at all-time points: 24 hpi (Wh: 4.1% ± 1.2; BA.5: 9.8% ± 2.2), 48 hpi (Wh: 38.3% ± 4.7; BA.5: 24.7% ± 1.9), and 72 hpi (Wh: 76.9% ± 4.0; BA.5: 53.9% ± 5.3). Similarly, viral load levels (RNA copies/mL) in Huh7.5 culture supernatants increased from 24 hpi to 72 hpi for both variants, with significant differences observed at 24 hpi (Wh: (1.55 ± 0.30) × 10^6^; BA.5: (7.53 ± 1.94) × 10^7^), 48 hpi (Wh: (2.61 ± 0.82) × 10^7^; BA.5: (9.64 ± 1.54) × 10^8^), and 72 hpi (Wh: (5.50 ± 0.39) × 10^8^; BA.5: (1.48 ± 0.21) × 10^9^). Confocal microscopy further confirmed SARS-CoV-2 infection in Huh7.5 cells (**Figure 1C**). These findings demonstrate that SARS-CoV-2 efficiently infects Huh7.5 cells, revealing differences in replication kinetics between the Wh and BA.5 variants.

To assess the infectivity of viral particles released into the Huh7.5 culture supernatants, samples collected at 24, 48, and 72 hpi were used to infect Vero E6 cells, following a previously described protocol. Infectious titers of SARS-CoV-2 in Vero E6 monolayers were determined at 72 hpi and expressed as PFU/mL. Results confirmed the infectivity of viral particles released from Huh7.5 cells and revealed variant-dependent differences in infection titers. Specifically, the viral titer obtained at 72 hpi for Vero E6 monolayers infected with supernatants collected at 24 and 48 hpi was (1.09 ± 0.10) × 10^5^ PFU/mL for Wh and (1.09 ± 0.10) × 10^8^ PFU/mL for BA.5. However, no differences were observed in viral titers when Vero E6 cells were infected with Huh7.5 supernatants collected at 72 hpi, with both variants yielding titers of (1.09 ± 0.10) × 10^8^ PFU/mL (**Figure 1D**).

In summary, both the Wh and BA.5 variants efficiently infect and replicate in hepatocytes, suggesting that hepatocyte infection may contribute, at least in part, to liver injury observed in COVID-19 patients. Furthermore, the accelerated replication kinetics of the Omicron (BA.5) compared to the ancestral (Wh) variant in hepatocytes may be linked to differences in cellular entry efficiency.

### 3.2 ACE2 and TfR1 (CD71) expression are differentially modulated in a variant-dependent manner

Although ACE2 is the primary receptor for SARS-CoV-2 entry, viral particles have been detected in multiple organs regardless of ACE2 expression levels (Liao et al., 2024). Other molecules expressed in extrapulmonary tissues can mediate viral entry into cells. One such molecule, the human transferrin receptor (TfR1), is a ubiquitously expressed membrane protein that interacts with the SARS-CoV-2 Spike protein with high affinity and facilitates viral entry via the endocytic pathway (Luo et al., 2022). Given that Huh7.5 cells are susceptible to SARS-CoV-2 infection, we evaluated the expression levels of ACE2 and TfR1 in these cells before and after infection. Huh7.5 cells were infected with the Wh and BA.5 variants at a multiplicity of infection (MOI) of 0.05. After 72 hpi, ACE2 and TfR1 expression levels were measured using flow cytometry.

The results showed that, following infection with either variant, the expression of ACE2 and TfR1, measured as median fluorescence intensity (MFI), was significantly higher than in control cells. Specifically, ACE2 expression levels were: Mock: 106.5 ± 2.1; Wh: 151.0 ± 8.4; BA.5: 166.0 ± 0.1. Similarly, TfR1 expression levels were: Mock: 69.7 ± 1.5; Wh: 138.7 ± 8.1; BA.5: 170.7 ± 2.0 (**Figure 1E**). Moreover, BA.5 induced a significantly greater increase in TfR1 expression in Huh7.5 cells compared to Wh. However, ACE2 upregulation occurred to a similar extent following infection with both variants (**Figure 1E**).

These findings demonstrate that SARS-CoV-2 infection upregulates ACE2 and TfR1 levels in a variant-dependent manner. Although BA.5 exhibits a high affinity for hACE2, it also harbors multiple Spike (S) protein mutations that may alter its conformation and reduce S1/S2 cleavage efficiency by TMPRSS2. As a result, BA.5 may rely on a late endocytic pathway for cell entry (Erabi et al., 2024). The higher TfR1 expression levels observed at 72 hpi with BA.5 may reflect TfR1’s role in mediating viral entry via endocytosis, the primary route used by this variant to infect cells.

### 3.3 SARS-CoV-2 infection induces cell death in Huh7.5 cells via both caspase-dependent and caspase-independent pathways

The SARS-CoV-2 Wuhan (Wh) and BA.5 variants efficiently infect and replicate in hepatocytes, potentially inducing cytopathic effects that trigger cell death pathways and contribute to tissue damage. Liver abnormalities have been observed in individuals infected with SARS-CoV-2, with postmortem studies revealing histopathological alterations. Annexin V and 7-AAD staining were used to assess apoptosis and necrosis by flow cytometry at 24, 48, and 72 hpi.

**Figure 2:**
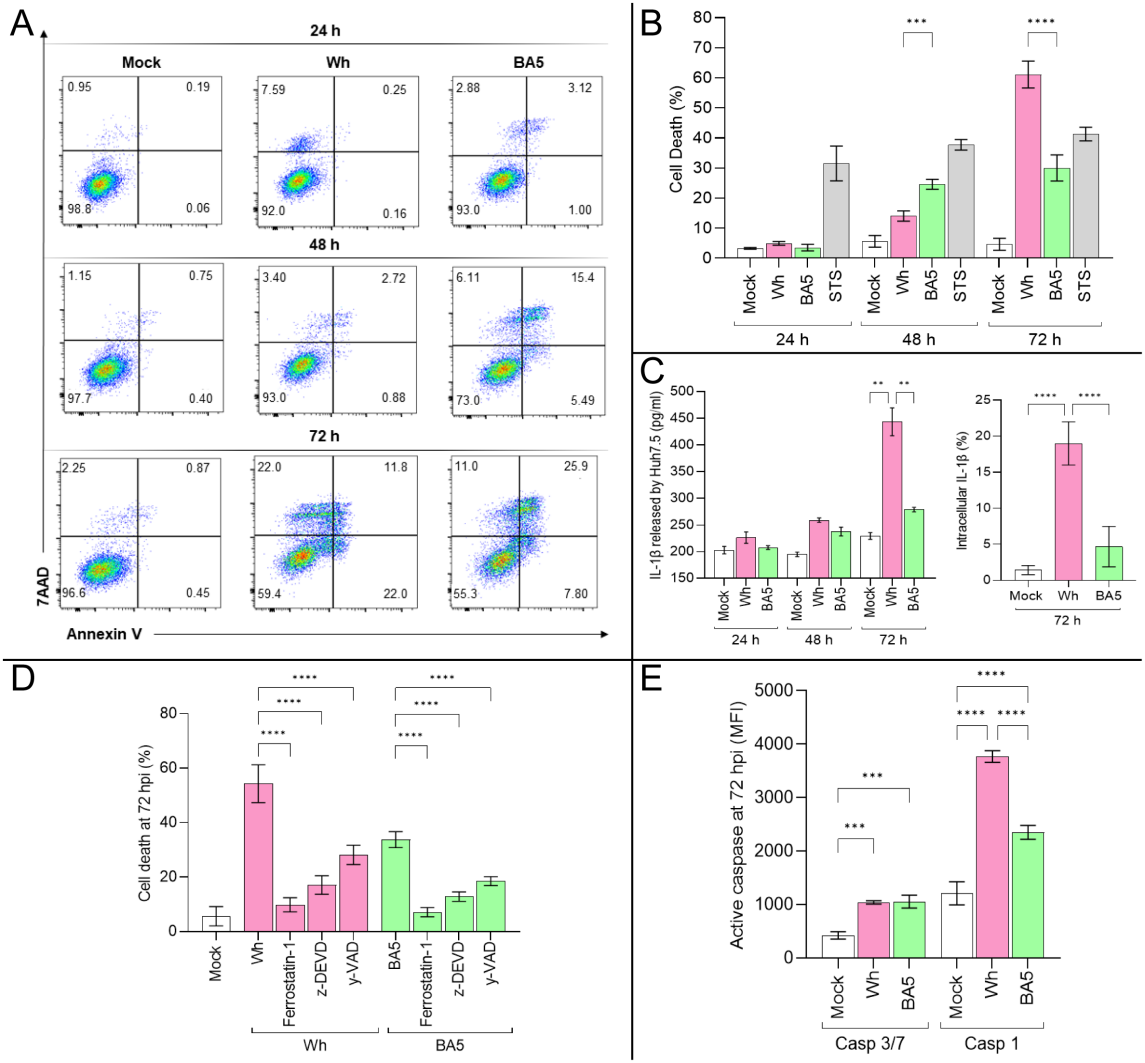
SARS-CoV-2 infection triggers regulated cell death pathways in Huh7.5 cells. **(A, B)** Quantification of cell death by Annexin V and 7-AAD staining at 24, 48, and 72 hpi in mock- and virus-infected cells. **(C)** IL-1β levels released to Huh7.5 culture supernatant and intracellular were measured at 72 hpi by ELISA and flow cytometry, respectively. **(D)** Effects of specific inhibitors of apoptosis (z-DEVD-FMK), pyroptosis (Y-VAD-FMK), and ferroptosis (ferrostatin-1) on virus-induced cell death at 72 hpi. **(E)** Activity of caspase-3/7 and caspase-1 at 72 hpi in infected cells. Data are presented as mean ± SD from three independent experiments. Statistical analysis was performed using one-way ANOVA with Tukey’s *post-hoc* test. **p<0.01, ***p<0.001, ****p<0.0001.

As shown in **Figure 2A** **, a** significant increase in Huh7.5 cell death was observed at 48 hpi (Mock: 6.5% ± 2.6; Wh: 14.0% ± 1.8; BA.5: 24.6% ± 1.7) and 72 hpi (Mock: 4.6% ± 2.0; Wh: 60.1% ± 4.8; BA.5: 30.0% ± 4.5). At 48 hpi, cell death in Wh-infected cells was significantly lower than in BA.5-infected cells. However, by 72 hpi, this trend reversed, with Wh-infected cells exhibiting significantly higher cell death levels than BA.5-infected cells (**Figure 2B**). Additionally, IL-1β production increased during viral replication in Huh7.5 cells. Notably, at 72 hpi, both extracellular and intracellular levels of IL-1β were significantly higher in Wh-infected cells compared to BA.5-infected cells (**Figure 2C**).

To investigate the involvement of specific cell death pathways, Huh7.5 cells were pretreated with inhibitors of caspase-3-dependent apoptosis (Z-DEVD), caspase-1-dependent pyroptosis (Z-YVAD), and ferroptosis (ferrostatin-1) before infection. Cell death levels at 72 hpi are shown in **Figure 2D**. Infected Huh7.5 cells showed 54.3% ± 6.9 (Wh) and 45.0% ± 4.7 (BA.5) cell death. Pretreatment with inhibitors significantly reduced cell death: Wh/ferrostatin-1: 9.8% ± 2.6; Wh/Z-DEVD: 17.1% ± 3.4; Wh/Z-YVAD: 28.2% ± 3.5; BA.5/ferrostatin-1: 14.0% ± 1.9; BA.5/Z-DEVD: 21.0% ± 3.4; BA.5/Z-YVAD: 28.5% ± 1.6.

The roles of caspase-dependent pathways were further explored by measuring caspase-3/7 and caspase-1 activity. Both were significantly elevated in infected cells compared to mock-infected controls, with caspase-1 activity showing a more pronounced increase (**Figure 2E**). These findings suggest that SARS-CoV-2 activates multiple cell death pathways in hepatocytes. Notably, ferroptosis, a caspase-independent mechanism, appears to contribute substantially to cell death regardless of the viral variant. The extent of SARS-CoV-2-induced regulated cell death in hepatocytes may vary by variant, likely reflecting differences in viral entry efficiency and replication kinetics.

### 3.4 SARS-CoV-2 infection induces mitochondrial dysfunction and mROS imbalance

Mitochondria play a crucial role in regulating multiple cell death pathways, including apoptotic and non-apoptotic mechanisms such as necroptosis, ferroptosis, pyroptosis, parthanatos, and paraptosis (Nguyen et al., 2023). Viruses, including SARS-CoV-2, can manipulate mitochondrial function, disrupting reactive oxygen species (ROS) homeostasis and contributing to cellular damage (Ren et al., 2020; Shang et al., 2021). Oxidative stress arises when excessive ROS production overwhelms the cellular antioxidant defenses, with mitochondrial respiration serving as a primary source of these free radicals.

**Figure 3:**
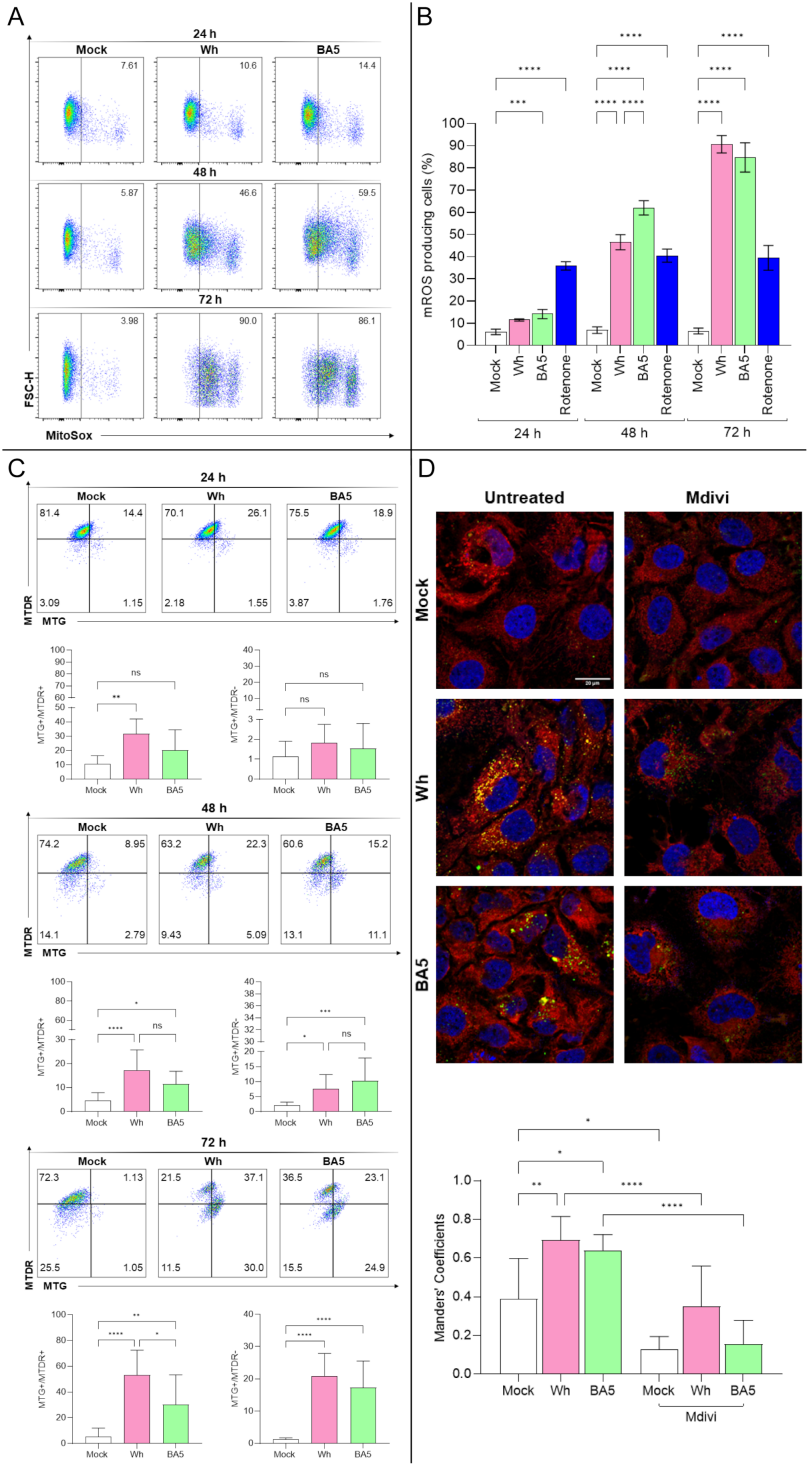
Mitochondrial dysfunction and mitophagy in SARS-CoV-2-infected hepatocytes. **(A, B)** Measurement of mitochondrial ROS (mROS) production in Huh7.5 cells infected with SARS-CoV-2 Wh or BA.5 at 24, 48, and 72 hpi using MitoSOX™ and flow cytometry. **(C)** Mitochondrial mass and membrane potential (ΔΨm) were assessed at 24, 48, and 72 hpi with MitoTracker™ Green (MTG) and Deep Red (MTDR) via flow cytometry. Representative dot plots are shown. **(D)** Confocal microscopy analysis of mitochondria–lysosome colocalization at 48 hpi. Mitochondria were stained with MitoTracker™ Red, lysosomes with LysoTracker™ Green, and nuclei with DAPI (blue). Colocalization was quantified using Manders’ overlap coefficient. Pretreatment with Mdivi-1 (50 µM) inhibited colocalization, indicating suppression of mitophagy. Data represent mean ± SD of three independent experiments. Statistical analysis was performed using one-way ANOVA with Tukey’s *post-hoc* test. *p<0.05, **p<0.01, ***p<0.001, ****p<0.0001. ns, not significant.

To assess the impact of SARS-CoV-2 infection on mitochondrial function, intracellular mitochondrial ROS (mROS) levels were measured at 24-, 48-, and 72-hpi in Huh7.5 cells infected with the Wh and BA.5 variants. Compared to mock-infected controls, both viral variants significantly increased mROS production at study time points. At 24 hpi, mROS levels were 6.1% ± 1.2 in mock-infected cells, 11.5% ± 0.5 in Wh-infected cells, and 14.2% ± 2.1 in BA.5-infected cells. At 48 hpi, the levels increased to 6.9% ± 1.5, 46.6% ± 3.4, and 62.0% ± 3.2, respectively. By 72 hpi, mROS production reached 6.5% ± 1.3 in mock-infected cells, 90.7% ± 3.9 in Wh-infected cells, and 84.7% ± 6.6 in BA.5-infected cells. At 48 hpi, mROS levels were significantly higher in BA.5-infected cells compared to Wh-infected cells. Still, this difference was no longer significant at 72 hpi, even when both variants substantially increased mROS production (**Figures 3A, B**).

To assess the impact of SARS-CoV-2 infection on mitochondrial function, Huh7.5 cells were analyzed by flow cytometry to evaluate mitochondrial mass (MitoTracker™ Green FM (MTG) and mitochondrial membrane potential –ΔΨm- (MitoTracker™ Deep Red FM (MTDR) following infection with either the ancestral (Wh) or omicron (BA.5) variants. Infected cells exhibited widespread mitochondrial fragmentation while largely maintaining ΔΨm (MTG+/MTDR+, indicating fragmented mitochondria with maintained membrane potential). Thus, at 24 hpi, the relative abundance of MTG+/MTDR+ was 10.6 ± 5.8% in mock-infected controls, 31.7 ± 10.4% in Wh-infected cells, and 20.1 ± 14.4% in BA.5-infected cells. The frequency of MTG+/MTDR− single-positive cells was 1.1 ± 0.8% (mock), 1.8 ± 0.9% (Wh), and 1.5 ± 1.3% (BA.5).

At 48 hpi, the abundance of double-positive cells was 4.6 ± 3.3% (mock), 19.4 ± 6.1% (Wh), and 11.5 ± 5.3% (BA.5), while MTG+/MTDR− cells increased to 2.1 ± 1.1% (mock), 7.7 ± 4.7% (Wh), and 10.3 ± 7.5% (BA.5).

Lastly, at 72 hpi, the proportion of double-positive cells rose to 5.2 ± 6.7% (mock), 53.1 ± 19.4% (Wh), and 30.4 ± 23.0% (BA.5), whereas MTG+/MTDR− single-positive cells were detected at 1.3 ± 0.4% (mock), 20.9 ± 7.0% (Wh), and 17.2 ± 8.3% (BA.5). These results indicate a time-dependent and variant-specific modulation of mitochondrial dynamics in infected cells. Notably, the accumulation of MTG+/MTDR− cells, indicative of mitochondrial depolarization, coincided with viral replication, suggesting that mitochondrial functionality may be compromised in a variant-dependent manner (**Figure 3C**).

Despite the marked increase in mROS production, mitochondrial fragmentation occurred without a complete loss of ΔΨm, suggesting the activation of compensatory mechanisms such as mitophagy. To investigate this possibility, the colocalization of mitochondria with lysosomal markers was analyzed by confocal microscopy at 48 hpi. SARS-CoV-2-infected cells exhibited a significant increase in mitochondrial-lysosomal colocalization compared to mock-infected controls, as quantified using Mander’s overlap coefficient. Notably, this colocalization was abolished when Huh7.5 cells were pretreated with the mitochondrial division inhibitor Mdivi, further supporting the involvement of mitochondrial fission in the activation of mitophagy (**Figure 3D**).

These findings reveal a dynamic mitochondrial response in Huh7.5 cells infected with SARS-CoV-2, characterized by an early increase in the population of cells with polarized mitochondria and preserved mitochondrial mass. This suggests a transient adaptive phase, possibly supporting viral replication by maintaining cellular bioenergetics and redox balance. However, at 48- and 72-hours post-infection, we observed an accumulation of depolarized mitochondria with preserved mass, accompanied by increased mitochondrial ROS (mROS), indicating progressive mitochondrial dysfunction. By 72 hours, features compatible with mitophagy were evident, suggesting an attempt by the cell to eliminate damaged mitochondria.

### 3.5 mROS scavenging impairs SARS-CoV-2 replication in Huh7.5 cells

To assess the contribution of mitochondrial reactive oxygen species (mROS) to SARS-CoV-2 replication, Huh7.5 cells were pretreated with either the mROS scavenger mitoTEMPO or the lipid peroxidation inhibitor ferrostatin-1, and subsequently infected with the ancestral Wuhan (Wh) or Omicron BA.5 variants. Viral replication was evaluated by quantifying viral RNA in culture supernatants using real-time PCR at 24, 48, and 72 hpi.

**Figure 4:**
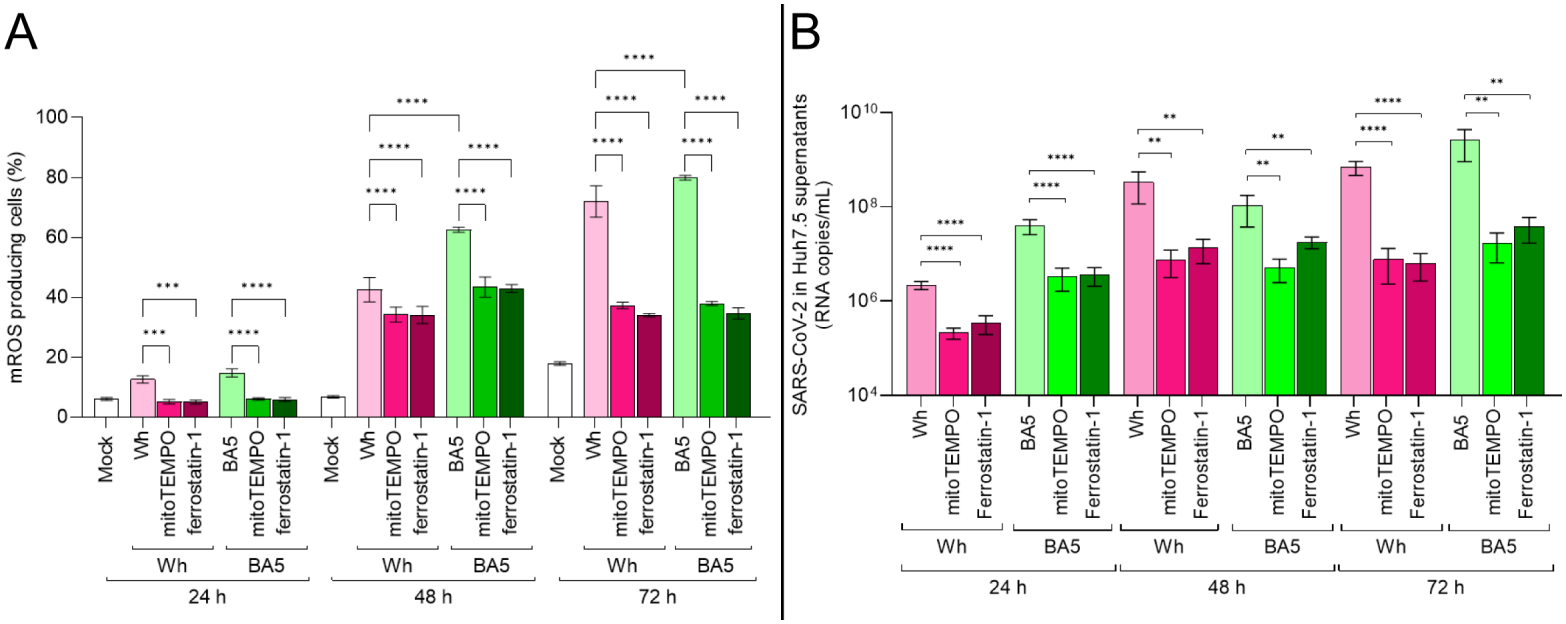
mROS scavenging impairs SARS-CoV-2 replication in hepatocytes. **(A)** mROS levels in infected Huh7.5 cells pretreated with MitoTEMPO (50 µM) or ferrostatin-1 (10 µM), measured at 24, 48, and 72 hpi by MitoSOX™ using flow cytometry. **(B)** Viral RNA copies in culture supernatants at each time point, quantified by RT-qPCR. Data show that antioxidant pretreatment significantly reduces mROS and viral replication in both Wh and BA.5 infections. Values are mean ± SD from three biological replicates. Statistical differences were evaluated using one-way ANOVA and Tukey’s *post-hoc* test. **p<0.01, ****p<0.0001.

As previously described (**Figure 3B**), SARS-CoV-2 infection is associated with a progressive increase in mROS levels from 24 to 72 hpi. As shown in **Figure 4A**, pretreatment with either antioxidant significantly reduced mROS accumulation by approximately twofold in infected cells, with similar effects observed for both viral variants. This reduction in oxidative stress was accompanied by a significant decrease in viral replication, with viral RNA levels in antioxidant-treated cells reduced by up to 100-fold compared to untreated controls (**Figure 4B**). This inhibitory effect was observed at study time points and was most pronounced at 48 and 72 hpi, coinciding with peak mROS production in untreated infections. Notably, mitoTEMPO exerted an inhibitory effect on viral replication comparable to that of ferrostatin-1, regardless of the viral variant.

Together, these findings indicate that SARS-CoV-2 replication in Huh7.5 cells is closely linked to mROS generation and that pharmacological scavenging of mitochondrial ROS significantly impairs viral replication in a variant-independent manner.

### 3.6 SARS-CoV-2 infection modulates the lipid metabolism in Huh7.5 cells

**Figure 5:**
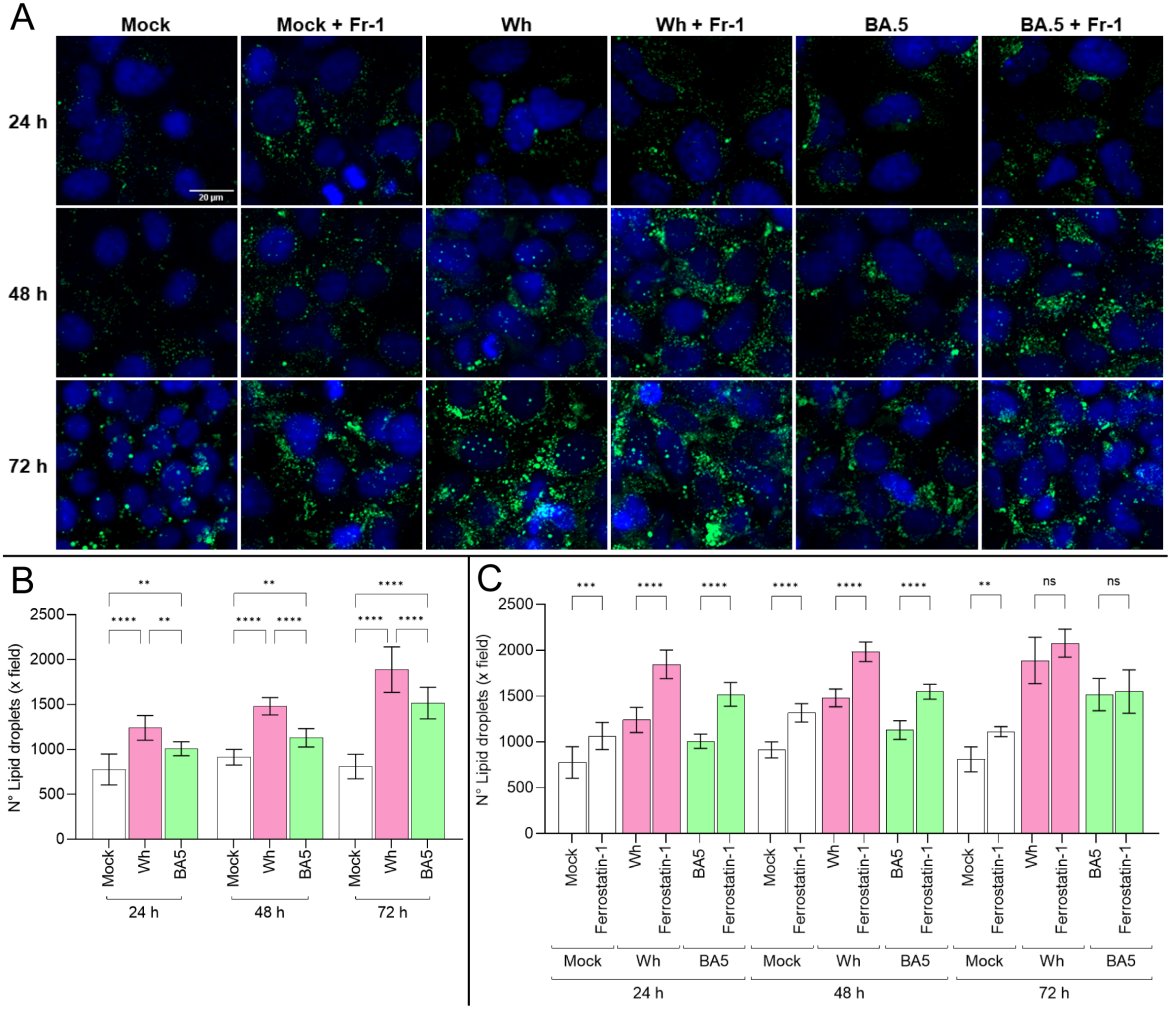
SARS-CoV-2 infection induces lipid droplet accumulation modulated by ferroptosis. **(A)** Representative confocal microscopy images of lipid droplet (LD) accumulation in Huh7.5 cells infected with Wh or BA.5 variants at 24, 48, and 72 hpi, stained with Bodipy™ 493/503 (green). Nuclei were stained with DAPI (blue). **(B, C)** Quantification of LD accumulation in untreated and ferrostatin-1-treated cells over time. Ferrostatin-1 pretreatment increased LD accumulation at early time points (24 and 48 hpi), but this effect was no longer observed at 72 hpi. Data represent mean ± SD from three independent experiments. Statistical analysis was performed using one-way ANOVA. **p<0.01, ***p<0.001, ****p<0.0001. ns, not significant.

Lipid synthesis, storage, and degradation are tightly regulated processes, with lipid droplets (LDs) functioning as dynamic organelles in infection, inflammation, and cellular homeostasis. To evaluate whether SARS-CoV-2 infection influences LD synthesis in Huh7.5 cells, cells were infected with the Wh and BA.5 variants (MOI=0.05). By using confocal microscopy, the LD accumulation was assessed at 24, 48, and 72 hpi. The results indicate that SARS-CoV-2 infection induces LD formation at 24 hpi, with sustained accumulation up to 72 hpi. The Wh variant induced significantly greater LD accumulation than BA.5 (**Figures 5A, B**).

Previous results demonstrated that ferroptosis contributes to regulated cell death in SARS-CoV-2-infected Huh7.5 cells. Moreover, disturbances in lipid metabolism are known to promote ferroptosis. Huh7.5 cells were pretreated with ferrostatin-1 for 24 hours before infection with either variant to explore a potential link between lipid droplet dynamics and ferroptosis. Under these conditions, LD accumulation was more pronounced in ferrostatin-1-treated cells at both 24 and 48 hpi for both variants. However, by 72 hpi, no differences in LD levels were observed between treated and untreated cells (**Figures 5A, C**).

These findings suggest that SARS-CoV-2 infection rapidly induces lipid droplet formation in Huh7.5 cells, with a variant-specific temporal profile that may be influenced by ferroptosis-related mechanisms. The transient effect of ferrostatin-1 on LD accumulation highlights a potential interplay between lipid metabolism and ferroptotic pathways during infection. Altogether, these results support the notion that SARS-CoV-2 modulates host lipid homeostasis, potentially contributing to viral replication and pathogenesis.

## 4 Discussion

The present study provides new insights into the direct effects of SARS-CoV-2 infection on hepatocytes, highlighting the contribution of mitochondrial dysfunction and ferroptosis to virus-induced liver injury. Although COVID-19 is primarily characterized as a respiratory illness, extrapulmonary manifestations—including hepatic dysfunction—are increasingly recognized (Bertolini et al., 2020; Wang et al., 2020; Dufour et al., 2022; Luo et al., 2022; Zhang et al., 2022; Quarleri and Delpino, 2024). Consistent with recent reports (Pradhan et al., 2023; Ko et al., 2025), our findings demonstrate that hepatocytes are permissive to SARS-CoV-2 infection. Histopathological and virological analyses of human liver biopsy and autopsy samples provide strong evidence for hepatic involvement in the clinical outcome of COVID-19 (Wanner et al., 2022; Maffia-Bizzozero et al., 2023; Oprinca et al., 2024; Rodriguez-Espada et al., 2024; Chen et al., 2025; Pita-Juarez et al., 2025; Sacco et al., 2025). Viral ancestral and Omicron variants modulate host cellular pathways to facilitate replication and promote cell death.

In line with a previous report (Foo et al., 2022), we observed a marked loss of mitochondrial membrane potential (Δψm) while total mitochondrial mass remained preserved, suggesting an early phase of mitochondrial stress and dysfunction. Then, an increased colocalization of mitochondria with lysosomes further supports the activation of mitophagy as a potential compensatory response to infection-induced mitochondrial stress, indicating that damaged mitochondria were selectively targeted for degradation. This sequence of events—depolarization with maintained mass followed by mitophagy—is consistent with previous studies demonstrating that SARS-CoV-2 disrupts mitochondrial homeostasis to modulate host cell responses. In particular, viral proteins such as ORF9b have been shown to localize to mitochondria and interfere with mitochondrial dynamics and antiviral signaling pathways, promoting mitochondrial depolarization and autophagic clearance (Gordon et al., 2020; Singh et al., 2020; Bhowal et al., 2023; Shoraka et al., 2023). Similar observations of mitophagy following Δψm dissipation have been reported in other viral contexts, underscoring the conserved nature of this stress response (Li et al., 2022). These alterations indicate mitochondrial stress and align with prior reports that describe mitochondrial manipulation as a hallmark of SARS-CoV-2 pathogenesis (Shang et al., 2021; Yu et al., 2023). The correlation between mROS levels and viral replication suggests a potential feed-forward loop wherein oxidative stress promotes viral propagation, possibly by altering antiviral signaling or supporting biosynthetic demands (Singh et al., 2020).

In support of this, we found that pharmacologic scavenging of mROS with mitoTEMPO or ferrostatin-1 significantly impaired SARS-CoV-2 replication in Huh7.5 cells, as shown by reduced viral RNA levels in the culture supernatants. These findings underscore the functional relevance of oxidative stress in sustaining efficient viral replication. Notably, mitoTEMPO, by specifically targeting mitochondrial superoxide, produced a slightly greater reduction in viral load than ferrostatin-1, particularly with the BA.5. This suggests that mitochondrial ROS may play a more direct role in viral replication than lipid peroxidation, which are interconnected (Guarnieri et al., 2024; Xie et al., 2024). Importantly, antioxidant pretreatment blunted the progressive increase in mROS observed during infection, supporting the idea that SARS-CoV-2 hijacks mitochondrial redox signaling to support its life cycle. These data support that antioxidant strategies, particularly those targeting mitochondrial ROS, could be therapeutic adjuncts to limit viral replication and protect hepatocellular integrity (Gain et al., 2022; Galli et al., 2022; Guarnieri et al., 2024; Xie et al., 2024).

In addition, infection induced lipid droplet (LD) accumulation and lipid peroxidation—hallmarks of ferroptosis, a regulated cell death (RCD) pathway characterized by iron-dependent lipid damage (Endale et al., 2023; Wang et al., 2023a).

Clinical evidence increasingly supports a role for ferroptosis in the development of COVID-19. Elevated serum ferritin and malondialdehyde (MDA), which are key markers of iron overload and lipid peroxidation, have been consistently observed in hospitalized and critically ill patients (Qiu et al., 2024). This reflects increased oxidative stress and ferroptotic cell death (Chen et al., 2022; Jankauskas et al., 2023; Peleman et al., 2023). At the same time, reduced levels of glutathione (GSH) and glutathione peroxidase 4 (GPX4) have been found in patient plasma and tissues exposed to SARS-CoV-2. These substances are essential for maintaining redox balance and preventing ferroptosis (Li et al., 2023; Zhao et al., 2024). Notably, losing GPX4 has been linked to greater disease severity and a higher risk of long COVID (Qiu et al., 2024). These findings are further supported by studies showing that serum from non-survivors of COVID-19 triggers lipid peroxidation and reduces GPX4 in human endothelial cells (Jankauskas et al., 2023). Together, these results suggest that ferroptosis may lead to multi-organ injury in COVID-19 and point to it as a possible treatment target.

Interestingly, we observed a progressive enlargement of LD in infected Huh7.5, becoming increasingly prominent from 24 to 72 hours post-infection, with the largest droplets correlating with the peak of cell death at 72 hours. This temporal pattern suggests that LD expansion may be linked to the intensification of oxidative stress and ferroptotic cell death. The temporal association between LD accumulation and ferroptosis inhibition with ferrostatin-1 suggests a functional interplay between disturbed lipid metabolism and ferroptotic cell death pathways in hepatocytes (Wu et al., 2020). These observations extend recent findings in lung, heart, and CNS tissues (Dias et al., 2020; Alizadeh Saghati et al., 2024; Jia and Han, 2024; Qiu et al., 2024) and suggest that ferroptosis may also contribute to COVID-19-associated liver pathology (Chen et al., 2022).

Single-cell RNA sequencing studies have shown ACE2 expression in 1–14% of cholangiocytes and 0.3–10% of hepatocytes (Ko et al., 2025). Hepatic ACE2 expression is elevated in patients with nonfibrotic MASH and shows a positive association with age, liver fat content, and fibroinflammatory markers. Similarly, increased hepatic ACE2 levels are observed in COVID-19 patients with liver injury (Cano et al., 2024; Jacobs et al., 2024; Rodriguez-Espada et al., 2024). Here, we demonstrate that ACE2 and TfR1 expression increased following infection, with variant-specific differences. While ACE2 remains the primary entry receptor for SARS-CoV-2, TfR1 has been shown to mediate viral entry independently of ACE2, especially in tissues with low ACE2 expression (Wang et al., 2023b; Liao et al., 2024). The upregulation of TfR1 could enhance viral entry through endocytosis while supporting ferroptosis via disturbed iron homeostasis (Habib et al., 2021). This dual role implies a plausible convergence of entry mechanisms and cell death pathways that may exacerbate liver injury (Wu et al., 2022). Together, in SARS-CoV-2-infected hepatocytes, ferroptosis appears to be triggered by a synergistic interaction between the rise of mROS, TfR1 overexpression, and lipid droplet accumulation, thus playing a role in hepatocellular damage during SARS-CoV-2 infection.

As previously reported (Scheuermann et al., 2023), differences in replication kinetics and cell death between variants were also observed. The ancestral strain showed faster replication and induced greater cell death at later time points than BA.5. These discrepancies may stem from differences in viral entry routes, membrane fusion efficiency, or immune evasion strategies. Importantly, both variants activated caspase-dependent (apoptosis and pyroptosis) and caspase-independent (ferroptosis) pathways, reflecting a multifaceted RCD response shaped by mitochondrial and immunometabolic stress (Vabret et al., 2020).

While pathway-specific chemical inhibitors helped delineate these responses, it is important to recognize the inherent complexity and overlap among regulated cell death (RCD) mechanisms (Eskander et al., 2025). For example, caspase-3 activity (apoptosis), caspase-1 activation, and IL-1β production (pyroptosis) were significantly elevated in infected cells. Nevertheless, inhibition of ferroptosis provided the most pronounced cytoprotective effect, reducing cell death by more than fivefold compared to untreated infected controls. Consequently, the data indicate that ferroptosis may play a prominent, though not exclusive, role, though additive or synergistic contributions from caspase-dependent pathways cannot be excluded.

Moreover, RCD pathways often share upstream signals, such as mitochondrial stress, ROS, and metabolic imbalance, that create extensive crosstalk (Marchi et al., 2012; Kist and Vucic, 2021). mROS, which was markedly increased during infection, can trigger ferroptosis and modulate caspase activity, illustrating the interdependency of these processes. Compensatory signaling may obscure the inhibition of one pathway when others remain active. Therefore, accurately quantifying the relative contribution of each RCD mechanism in the context of dynamic infection remains methodologically challenging, particularly in the absence of targeted genetic tools or single-cell resolution techniques.

Future studies employing time-resolved analyses, multi-parameter imaging, or integrative omics may better resolve the sequence and hierarchy of cell death events. Understanding this interplay is not only of mechanistic importance but may also inform therapeutic strategies to mitigate tissue damage by selectively modulating host responses.

Overall, this study underscores the relevance of regulated cell death, particularly ferroptosis, in SARS-CoV-2-infected hepatocytes and highlights the central role of mitochondrial dysfunction and oxidative stress in shaping disease outcomes. Considering the frequency of liver abnormalities in patients with COVID-19, our findings have significant implications for understanding the pathophysiology of extrapulmonary SARS-CoV-2 infection and identifying novel therapeutic targets. Pharmacologic modulation of mitochondrial ROS or ferroptosis may offer promising strategies to protect hepatic tissue during acute infection and reduce long-term sequelae in COVID-19 survivors.

Limitations of our study should be acknowledged. First, we employed the Huh7.5 hepatoma-derived cell line, which, while widely accepted as a hepatocyte model, does not fully capture the complexity of primary hepatocytes or the liver microenvironment. The transformed nature of these cells may alter mitochondrial behavior and stress responses, potentially limiting generalizability. Second, although we demonstrated upregulation of ACE2 and TfR1 and highlighted the functional relevance of mROS and ferroptosis, we did not examine the upstream signaling pathways in depth. Third, the absence of *in vivo* validation limits our ability to assess immune-mediated effects or multicellular interactions that occur in liver tissue during infection. Fourth, Bodipy 493/503 mainly detects neutral lipids and cannot differentiate oxidized lipid species. As a result, it might underestimate the level of lipid peroxidation. Furthermore, measuring GPX4 enzymatic activity directly or quantifying specific lipid peroxidation markers like malondialdehyde (MDA) or 4-hydroxynonenal (4-HNE) would offer stronger evidence for the role of ferroptosis. Finally, while we analyzed two viral variants, expanding the scope to include emerging lineages would enhance the translational value of our findings.

Despite these limitations, our study provides novel insights into the mechanisms underlying SARS-CoV-2-induced liver injury and offers a foundation for future investigations using primary hepatocytes, organoid models, or *in vivo* systems. A deeper understanding of how viral replication intersects with host stress responses and immune signaling will be essential for developing effective interventions to prevent or treat liver damage in COVID-19 and related viral diseases.

## Data Availability

The raw data supporting the conclusions of this article will be made available by the authors, without undue reservation.

## References

[B1] Alizadeh Saghati A. Sharifi Z. Hatamikhah M. Salimi M. Talkhabi M. (2024). Unraveling the relevance of SARS-Cov-2 infection and ferroptosis within the heart of COVID-19 patients. Heliyon 10, e36567. doi: 10.1016/j.heliyon.2024.e36567, PMID: 39263089 PMC11388749

[B2] Bartolomeo C. S. Lemes R. M. R. Morais R. L. Pereria G. C. Nunes T. A. Costa A. J.. (2022). SARS-CoV-2 infection and replication kinetics in different human cell types: The role of autophagy, cellular metabolism and ACE2 expression. Life Sci. 308, 120930. doi: 10.1016/j.lfs.2022.120930, PMID: 36075471 PMC9444585

[B3] Bertolini A. van de Peppel I. P. Bodewes F. Moshage H. Fantin A. Farinati F.. (2020). Abnormal liver function tests in patients with COVID-19: relevance and potential pathogenesis. Hepatology 72, 1864–1872. doi: 10.1002/hep.31480, PMID: 32702162 PMC7404414

[B4] Bhowal C. Ghosh S. Ghatak D. De R. (2023). Pathophysiological involvement of host mitochondria in SARS-CoV-2 infection that causes COVID-19: a comprehensive evidential insight. Mol. Cell Biochem. 478, 1325–1343. doi: 10.1007/s11010-022-04593-z, PMID: 36308668 PMC9617539

[B5] Cano L. Desquilles L. Ghukasyan G. Angenard G. Landreau C. Corlu A.. (2024). SARS-CoV-2 receptor ACE2 is upregulated by fatty acids in human MASH. JHEP Rep. 6, 100936. doi: 10.1016/j.jhepr.2023.100936, PMID: 38074511 PMC10698276

[B6] Chen S. Zhang Y. Ashuo A. Song S. Yuan L. Wang W.. (2025). Combination of spatial transcriptomics analysis and retrospective study reveals liver infection of SARS-COV-2 is associated with clinical outcomes of COVID-19. EBioMedicine 111, 105517. doi: 10.1016/j.ebiom.2024.105517, PMID: 39709771 PMC11732063

[B7] Chen Y. Xu Y. Zhang K. Shen L. Deng M. (2022). Ferroptosis in COVID-19-related liver injury: A potential mechanism and therapeutic target. Front. Cell Infect. Microbiol. 12. doi: 10.3389/fcimb.2022.922511, PMID: 35967872 PMC9363633

[B8] D'Avila H. Lima C. N. R. Rampinelli P. G. Mateus L. C. O. Sousa Silva R. V. Correa J. R.. (2024). Lipid metabolism modulation during SARS-coV-2 infection: A spotlight on extracellular vesicles and therapeutic prospects. Int. J. Mol. Sci. 25 (1), 640. doi: 10.3390/ijms25010640, PMID: 38203811 PMC10778989

[B9] Dias S. S. G. Soares V. C. Ferreira A. C. Sacramento C. Q. Fintelman-Rodrigues N. Temerozo J. R.. (2020). Lipid droplets fuel SARS-CoV-2 replication and production of inflammatory mediators. PloS Pathog. 16, e1009127. doi: 10.1371/journal.ppat.1009127, PMID: 33326472 PMC7773323

[B10] Dufour J. F. Marjot T. Becchetti C. Tilg H. (2022). COVID-19 and liver disease. Gut 71, 2350–2362. doi: 10.1136/gutjnl-2021-326792, PMID: 35701093

[B11] Endale H. T. Tesfaye W. Mengstie T. A. (2023). ROS induced lipid peroxidation and their role in ferroptosis. Front. Cell Dev. Biol. 11. doi: 10.3389/fcell.2023.1226044, PMID: 37601095 PMC10434548

[B12] Erabi G. Faridzadeh A. Parvin A. Deravi N. Rahmanian M. Fathi M.. (2024). SARS-CoV-2 Omicron (BA.4, BA.5) variant: Lessons learned from a new variant during the COVID-19 pandemic. Health Sci. Rep. 7, e1873. doi: 10.1002/hsr2.1873, PMID: 38332930 PMC10851086

[B13] Eskander G. Abdelhamid S. G. Wahdan S. A. Radwan S. M. (2025). Insights on the crosstalk among different cell death mechanisms. Cell Death Discov. 11, 56. doi: 10.1038/s41420-025-02328-9, PMID: 39929794 PMC11811070

[B14] Foo J. Bellot G. Pervaiz S. Alonso S. (2022). Mitochondria-mediated oxidative stress during viral infection. Trends Microbiol. 30, 679–692. doi: 10.1016/j.tim.2021.12.011, PMID: 35063304

[B15] Fratta Pasini A. M. Stranieri C. Girelli D. Busti F. Cominacini L. (2021). Is ferroptosis a key component of the process leading to multiorgan damage in COVID-19? Antioxidants (Basel) 10 (11), 1677. doi: 10.3390/antiox10111677, PMID: 34829548 PMC8615234

[B16] Gain C. Song S. Angtuaco T. Satta S. Kelesidis T. (2022). The role of oxidative stress in the pathogenesis of infections with coronaviruses. Front. Microbiol. 13. doi: 10.3389/fmicb.2022.1111930, PMID: 36713204 PMC9880066

[B17] Galli F. Marcantonini G. Giustarini D. Albertini M. C. Migni A. Zatini L.. (2022). How aging and oxidative stress influence the cytopathic and inflammatory effects of SARS-coV-2 infection: the role of cellular glutathione and cysteine metabolism. Antioxidants (Basel) 11:1366. doi: 10.3390/antiox11071366, PMID: 35883857 PMC9311797

[B18] Gordon D. E. Jang G. M. Bouhaddou M. Xu J. Obernier K. White K. M.. (2020). A SARS-CoV-2 protein interaction map reveals targets for drug repurposing. Nature 583, 459–468. doi: 10.1038/s41586-020-2286-9, PMID: 32353859 PMC7431030

[B19] Guarnieri J. W. Lie T. Albrecht Y. E. S. Hewin P. Jurado K. A. Widjaja G. A.. (2024). Mitochondrial antioxidants abate SARS-COV-2 pathology in mice. Proc. Natl. Acad. Sci. U.S.A. 121, e2321972121. doi: 10.1073/pnas.2321972121, PMID: 39008677 PMC11287122

[B20] Habib H. M. Ibrahim S. Zaim A. Ibrahim W. H. (2021). The role of iron in the pathogenesis of COVID-19 and possible treatment with lactoferrin and other iron chelators. BioMed. Pharmacother. 136, 111228. doi: 10.1016/j.biopha.2021.111228, PMID: 33454595 PMC7836924

[B21] Jacobs A. K. Morley S. D. Samuel K. Morgan K. Boswell L. Kendall T. J.. (2024). Hepatic angiotensin-converting enzyme 2 expression in metabolic dysfunction-associated steatotic liver disease and in patients with fatal COVID-19. World J. Gastroenterol. 30, 3705–3716. doi: 10.3748/wjg.v30.i31.3705, PMID: 39192998 PMC11346159

[B22] Jankauskas S. S. Kansakar U. Sardu C. Varzideh F. Avvisato R. Wang X.. (2023). COVID-19 causes ferroptosis and oxidative stress in human endothelial cells. Antioxidants (Basel) 12 (2), 326. doi: 10.3390/antiox12020326, PMID: 36829885 PMC9952002

[B23] Jia F. Han J. (2024). COVID-19 related neurological manifestations in Parkinson's disease: has ferroptosis been a suspect? Cell Death Discov. 10, 146. doi: 10.1038/s41420-024-01915-6, PMID: 38503730 PMC10951317

[B24] Kist M. Vucic D. (2021). Cell death pathways: intricate connections and disease implications. EMBO J. 40, e106700. doi: 10.15252/embj.2020106700, PMID: 33439509 PMC7917554

[B25] Ko C. Cheng C. C. Mistretta D. Ambike S. Sacherl J. Velkov S.. (2025). SARS-coV-2 productively infects human hepatocytes and induces cell death. J. Med. Virol. 97, e70156. doi: 10.1002/jmv.70156, PMID: 39760326 PMC11702151

[B26] Li Q. Chen Z. Zhou X. Li G. Zhang C. Yang Y. (2023). Ferroptosis and multi-organ complications in COVID-19: mechanisms and potential therapies. Front. Genet. 14. doi: 10.3389/fgene.2023.1187985, PMID: 37303950 PMC10250669

[B27] Li Y. Wu K. Zeng S. Zou L. Li X. Xu C.. (2022). The role of mitophagy in viral infection. Cells 11 (4), 711. doi: 10.3390/cells11040711, PMID: 35203359 PMC8870278

[B28] Liao Z. Wang C. Tang X. Yang M. Duan Z. Liu L.. (2024). Human transferrin receptor can mediate SARS-CoV-2 infection. Proc. Natl. Acad. Sci. U.S.A. 121, e2317026121. doi: 10.1073/pnas.2317026121, PMID: 38408250 PMC10927525

[B29] Luo M. Ballester M. P. Soffientini U. Jalan R. Mehta G. (2022). SARS-CoV-2 infection and liver involvement. Hepatol. Int. 16, 755–774. doi: 10.1007/s12072-022-10364-1, PMID: 35767172 PMC9243815

[B30] Maffia-Bizzozero S. Cevallos C. Lenicov F. R. Freiberger R. N. Lopez C. A. M. Guano Toaquiza A.. (2023). Viable SARS-CoV-2 Omicron sub-variants isolated from autopsy tissues. Front. Microbiol. 14. doi: 10.3389/fmicb.2023.1192832, PMID: 37283920 PMC10240073

[B31] Manders E. M. M. Verbeek F. J. Aten J. A. (1993). Measurement of co-localization of objects in dual-colour confocal images. J. Microsc 169, 375–382. doi: 10.1111/j.1365-2818.1993.tb03313.x, PMID: 33930978

[B32] Marchi S. Giorgi C. Suski J. M. Agnoletto C. Bononi A. Bonora M.. (2012). Mitochondria-ros crosstalk in the control of cell death and aging. J. Signal Transduct 2012, 329635. doi: 10.1155/2012/329635, PMID: 22175013 PMC3235816

[B33] Muhoberac B. B. (2020). What can cellular redox, iron, and reactive oxygen species suggest about the mechanisms and potential therapy of COVID-19? Front. Cell Infect. Microbiol. 10. doi: 10.3389/fcimb.2020.569709, PMID: 33381464 PMC7767833

[B34] Nguyen T. T. Wei S. Nguyen T. H. Jo Y. Zhang Y. Park W.. (2023). Mitochondria-associated programmed cell death as a therapeutic target for age-related disease. Exp. Mol. Med. 55, 1595–1619. doi: 10.1038/s12276-023-01046-5, PMID: 37612409 PMC10474116

[B35] Ojeda D. S. Grasso D. Urquiza J. Till A. Vaccaro M. I. Quarleri J. (2018). Cell death is counteracted by mitophagy in HIV-productively infected astrocytes but is promoted by inflammasome activation among non-productively infected cells. Front. Immunol. 9. doi: 10.3389/fimmu.2018.02633, PMID: 30515154 PMC6255949

[B36] Oprinca G. C. Mohor C. I. Bereanu A. S. Oprinca-Muja L. A. Bogdan-Duica I. Fleaca S. R.. (2024). Detection of SARS-coV-2 viral genome and viral nucleocapsid in various organs and systems. Int. J. Mol. Sci. 25 (11), 5755. doi: 10.3390/ijms25115755, PMID: 38891942 PMC11172220

[B37] Peleman C. Van Coillie S. Ligthart S. Choi S. M. De Waele J. Depuydt P.. (2023). Ferroptosis and pyroptosis signatures in critical COVID-19 patients. Cell Death Differ 30, 2066–2077. doi: 10.1038/s41418-023-01204-2, PMID: 37582864 PMC10482958

[B38] Pita-Juarez Y. Karagkouni D. Kalavros N. Melms J. C. Niezen S. Delorey T. M.. (2025). A single-nucleus and spatial transcriptomic atlas of the COVID-19 liver reveals topological, functional, and regenerative organ disruption in patients. Genome Biol. 26, 56. doi: 10.1186/s13059-025-03499-5, PMID: 40087773 PMC11907808

[B39] Pradhan S. Rouster S. D. Blackard J. T. Dean G. E. Sherman K. E. (2023). Replication and injury associated with SARS-coV-2 in cultured hepatocytes. Pathog. Immun. 8, 59–73. doi: 10.20411/pai.v8i2.648, PMID: 38361525 PMC10868721

[B40] Proal A. D. VanElzakker M. B. Aleman S. Bach K. Boribong B. P. Buggert M.. (2023). SARS-CoV-2 reservoir in post-acute sequelae of COVID-19 (PASC). Nat. Immunol. 24, 1616–1627. doi: 10.1038/s41590-023-01601-2, PMID: 37667052

[B41] Qiu B. Zandkarimi F. Saqi A. Castagna C. Tan H. Sekulic M.. (2024). Fatal COVID-19 pulmonary disease involves ferroptosis. Nat. Commun. 15, 3816. doi: 10.1038/s41467-024-48055-0, PMID: 38769293 PMC11106344

[B42] Quarleri J. Delpino M. V. (2024). Molecular mechanisms underlying SARS-CoV-2 hepatotropism and liver damage. World J. Hepatol. 16, 1–11. doi: 10.4254/wjh.v16.i1.1, PMID: 38313242 PMC10835487

[B43] Ren Z. Zhang X. Ding T. Zhong Z. Hu H. Xu Z.. (2020). Mitochondrial dynamics imbalance: A strategy for promoting viral infection. Front. Microbiol. 11. doi: 10.3389/fmicb.2020.01992, PMID: 32973718 PMC7472841

[B44] Rodriguez-Espada A. Salgado-de la Mora M. Rodriguez-Paniagua B. M. Limon-de la Rosa N. Martinez-Gutierrez M. I. Pastrana-Brandes S.. (2024). Histopathological impact of SARS-CoV-2 on the liver: Cellular damage and long-term complications. World J. Gastroenterol. 30, 2866–2880. doi: 10.3748/wjg.v30.i22.2866, PMID: 38947288 PMC11212712

[B45] Sacco M. A. Gualtieri S. Princi A. Verrina M. C. Carbone A. Tarda L.. (2025). Investigating the post-mortem risk of transmission of SARS-coV-2 virus in cadaveric tissues: A systematic review of the literature. Microorganisms 13 (2),284. doi: 10.3390/microorganisms13020284, PMID: 40005651 PMC11858283

[B46] Scheuermann S. E. Goff K. Rowe L. A. Beddingfield B. J. Maness N. J. (2023). Real-time analysis of SARS-coV-2-induced cytolysis reveals distinct variant-specific replication profiles. Viruses 15 (9), 1937. doi: 10.3390/v15091937, PMID: 37766343 PMC10537736

[B47] Shang C. Liu Z. Zhu Y. Lu J. Ge C. Zhang C.. (2021). SARS-coV-2 causes mitochondrial dysfunction and mitophagy impairment. Front. Microbiol. 12. doi: 10.3389/fmicb.2021.780768, PMID: 35069483 PMC8770829

[B48] Shoraka S. Samarasinghe A. E. Ghaemi A. Mohebbi S. R. (2023). Host mitochondria: more than an organelle in SARS-CoV-2 infection. Front. Cell Infect. Microbiol. 13. doi: 10.3389/fcimb.2023.1228275, PMID: 37692170 PMC10485703

[B49] Singh K. K. Chaubey G. Chen J. Y. Suravajhala P. (2020). Decoding SARS-CoV-2 hijacking of host mitochondria in COVID-19 pathogenesis. Am. J. Physiol. Cell Physiol. 319, C258–C267. doi: 10.1152/ajpcell.00224.2020, PMID: 32510973 PMC7381712

[B50] Sviercz F. Jarmoluk P. Godoy Coto J. Cevallos C. Freiberger R. N. Lopez C. A. M.. (2024). The abortive SARS-CoV-2 infection of osteoclast precursors promotes their differentiation into osteoclasts. J. Med. Virol. 96, e29597. doi: 10.1002/jmv.29597, PMID: 38587211

[B51] Vabret N. Britton G. J. Gruber C. Hegde S. Kim J. Kuksin M.. (2020). Immunology of COVID-19: current state of the science. Immunity 52, 910–941. doi: 10.1016/j.immuni.2020.05.002, PMID: 32505227 PMC7200337

[B52] Wang Y. Liu S. Liu H. Li W. Lin F. Jiang L.. (2020). SARS-CoV-2 infection of the liver directly contributes to hepatic impairment in patients with COVID-19. J. Hepatol. 73, 807–816. doi: 10.1016/j.jhep.2020.05.002, PMID: 32437830 PMC7211738

[B53] Wang B. Wang Y. Zhang J. Hu C. Jiang J. Li Y.. (2023a). ROS-induced lipid peroxidation modulates cell death outcome: mechanisms behind apoptosis, autophagy, and ferroptosis. Arch. Toxicol. 97, 1439–1451. doi: 10.1007/s00204-023-03476-6, PMID: 37127681

[B54] Wang X. Wen Z. Cao H. Luo J. Shuai L. Wang C.. (2023b). Transferrin receptor protein 1 cooperates with mGluR2 to mediate the internalization of rabies virus and SARS-coV-2. J. Virol. 97, e0161122. doi: 10.1128/jvi.01611-22, PMID: 36779763 PMC9972945

[B55] Wanner N. Andrieux G. Badia I. M. P. Edler C. Pfefferle S. Lindenmeyer M. T.. (2022). Molecular consequences of SARS-CoV-2 liver tropism. Nat. Metab. 4, 310–319. doi: 10.1038/s42255-022-00552-6, PMID: 35347318 PMC8964418

[B56] Wissler Gerdes E. O. Vanichkachorn G. Verdoorn B. P. Hanson G. J. Joshi A. Y. Murad M. H.. (2022). Role of senescence in the chronic health consequences of COVID-19. Transl. Res. 241, 96–108. doi: 10.1016/j.trsl.2021.10.003, PMID: 34695606 PMC8532377

[B57] Wu Y. Jiao H. Yue Y. He K. Jin Y. Zhang J.. (2022). Ubiquitin ligase E3 HUWE1/MULE targets transferrin receptor for degradation and suppresses ferroptosis in acute liver injury. Cell Death Differ 29, 1705–1718. doi: 10.1038/s41418-022-00957-6, PMID: 35260822 PMC9433446

[B58] Wu J. Song S. Cao H. C. Li L. J. (2020). Liver diseases in COVID-19: Etiology, treatment and prognosis. World J. Gastroenterol. 26, 2286–2293. doi: 10.3748/wjg.v26.i19.2286, PMID: 32476793 PMC7243650

[B59] Xie J. Yuan C. Yang S. Ma Z. Li W. Mao L.. (2024). The role of reactive oxygen species in severe acute respiratory syndrome coronavirus 2 (SARS-COV-2) infection-induced cell death. Cell Mol. Biol. Lett. 29, 138. doi: 10.1186/s11658-024-00659-6, PMID: 39516736 PMC11549821

[B60] Yu H. Yang L. Han Z. Zhou X. Zhang Z. Sun T.. (2023). SARS-CoV-2 nucleocapsid protein enhances the level of mitochondrial reactive oxygen species. J. Med. Virol. 95, e29270. doi: 10.1002/jmv.29270, PMID: 38047459

[B61] Yuan C. Ma Z. Xie J. Li W. Su L. Zhang G.. (2023). The role of cell death in SARS-CoV-2 infection. Signal Transduct Target Ther. 8, 357. doi: 10.1038/s41392-023-01580-8, PMID: 37726282 PMC10509267

[B62] Zhang J. Zhao D. Hu J. Huang X. Gu Q. Tao Z. (2022). Hepatic dysfunctions in COVID-19 patients infected by the omicron variant of SARS-CoV-2. Front. Public Health 10. doi: 10.3389/fpubh.2022.1049006, PMID: 36466505 PMC9716022

[B63] Zhao W. Wang S. Han Y. Zhang H. Cao J. Dong S.. (2024). Role of ferroptosis in the progression of COVID-19 and the development of long COVID. Curr. Med. Chem. 32, 4324–4342. doi: 10.2174/0109298673281662231208102354, PMID: 38310391

